# Temporomandibular joint arthrocentesis and its effect on articular capsular width: a clinical ultrasonographic study

**DOI:** 10.1186/s12903-026-08803-8

**Published:** 2026-06-01

**Authors:** Onur Sahar, Sema Kaya

**Affiliations:** 1 Department of Oral and Maxillofacial Surgery, Faculty of Dentistry, Van Yuzuncu Yil University, Van, Turkey; 2 Department of Oral and Maxillofacial Radiology, Faculty of Dentistry, Van Yuzuncu Yil University, Van, Turkey

**Keywords:** Temporomandibular joint, Temporomandibular disorders, Articular capsular width, Ultrasonographic

## Abstract

**Background:**

The efficacy of arthrocentesis in temporomandibular joint (TMJ) disorders is well established, but its effect on articular capsular width is unclear. The purpose of this study was to evaluate changes in capsular width associated with TMJ arthrocentesis using ultrasonography.

**Methods:**

This prospective cohort study included individuals presenting with temporomandibular disorders (TMD) to Van Yuzuncu Yil University. Inclusion criteria were systemically healthy adults who provided informed consent and had intra-articular TMD persisting for ≥ 6 months despite conservative management. Statistical analyses included Mann-Whitney U test, Wilcoxon signed-rank test, and Friedman test for group comparisons, with significance at *p* < 0.05. Spearman correlation analysis was performed to assess associations between changes in capsular width and clinical outcomes.

**Results:**

The study sample consisted of 27 subjects (16 females, 59.3%) with a mean age of 41.6 ± 13.0 years. Ultrasonographic analysis revealed a significant reduction in capsular width on the affected side over time (*p* < 0.001), whereas no significant change was observed on the non-affected side (*p* = 0.4). The mean capsular width on the affected side decreased from 2.73 ± 0.29 mm at baseline (T0, pre-treatment) to 2.46 ± 0.30 mm at three months after arthrocentesis (T1) and 2.29 ± 0.33 mm at six months after arthrocentesis (T2). Between-group comparisons showed no significant difference at T0 (*p* = 0.3) or at T1 (*p* = 0.1), but at T2, the affected side demonstrated a significantly smaller capsular width compared with the non-affected side (*p* < 0.001).

**Conclusions:**

The findings suggest that arthrocentesis was associated with a reduction in capsular width on the treated side, concurrent with improvements in pain and mandibular function.

**Trial registration:**

ClinicalTrials.gov (NCT07553078), registered on April 15, 2026. Retrospectively registered.

## Introduction

The temporomandibular joint (TMJ) is a paired ellipsoid synovial joint formed between the mandibular condyle and the temporal bone. Like other synovial joints, it consists of a disc, bone, fibrous capsule, synovial fluid, synovial membrane, and ligaments. However, the most distinctive feature that differentiates this joint from other synovial joints is that its articular surfaces are covered with fibrocartilage instead of hyaline cartilage [1]. As a synovial joint, the TMJ is prone to various disorders, including inflammatory and degenerative diseases. These pathological changes may impair normal joint function and contribute to pain and restricted mandibular movement [2].

Recent advances in the understanding of temporomandibular disorders (TMD) have highlighted their heterogeneous and multifactorial nature. TMD comprises a group of musculoskeletal and neuromuscular conditions involving the TMJ, masticatory muscles, and associated structures. While internal derangement—defined as disc-condyle-fossa misalignment—was historically emphasized as a primary source of symptoms, current evidence indicates that pain and functional limitation are more closely related to a complex interplay of factors, including synovial inflammation, joint effusion, capsular width, and different types of disc displacement. In patients with intra-articular TMD presenting with limited mandibular movement, arthrocentesis is often considered a first-line minimally invasive treatment. The procedure facilitates lavage of the upper joint compartment, reduces inflammatory mediators, and improves joint mobility and function [3, 4].

Historically, the assessment of TMJ disorders primarily relied on plain radiographs, followed by more detailed evaluations using techniques such as arthrography and computed tomography [5]. However, due to the invasive nature of these techniques and the associated radiation exposure, magnetic resonance imaging and ultrasonography have become widely used imaging modalities in recent years [6–8]. Ultrasonography is a low-cost, easily accessible imaging modality that can be performed in most outpatient clinics. It has a short procedure time and carries no known side effects or risks. Furthermore, direct interaction with the patient during ultrasonography allows for more precise localization of pain. In addition, the ultrasound probe can be used as a palpable tool to detect clinical findings such as real-time crepitus, clicking sounds, joint movements, and sudden shifts. The high patient comfort, dynamic imaging capability, and non-invasive nature of ultrasonography enhance its clinical utility in the evaluation of TMD. In the context of the present study, ultrasonography was preferred due to its accessibility, non-invasive nature, and suitability for repeated follow-up assessments without radiation exposure. However, ultrasonography is known to have certain limitations, including operator dependency and limited ability to visualize deeper joint structures compared to magnetic resonance imaging [9–14].

The aim of this study was to evaluate changes in intra-articular capsular width following TMJ arthrocentesis using ultrasonography and to explore their relationship with clinical outcomes. The study hypothesis was that significant changes in capsular width would be observed after the procedure. Accordingly, capsular width, pain levels, maximum incisal opening (MIO), and lateral and protrusive mandibular movements were assessed over a six-month follow-up period. Limited prospective clinical evidence exists regarding longitudinal changes in TMJ capsular width assessed by ultrasonography after arthrocentesis.

## Materials and methods

### Study design and ethical approval

This investigation was designed as a prospective cohort study evaluating changes in capsular width following TMJ arthrocentesis. The study was conducted in accordance with medical protocols and the principles of the Declaration of Helsinki. Ethical approval was obtained from the Clinical Research Ethics Committee of Van Yuzuncu Yil University (approval no. 2025/02–07, dated February 28, 2025). Patients presenting with TMD to the Oral and Maxillofacial Surgery and Dentomaxillofacial Radiology clinics at Van Yuzuncu Yil University between February 2025 and September 2025 were consecutively screened and enrolled.

### Participants and diagnostic criteria

All patients underwent a standardized clinical examination according to the Diagnostic Criteria for Temporomandibular Disorders (DC/TMD). Only patients diagnosed with intra-articular TMD, specifically disc displacement with or without reduction according to the DC/TMD criteria, were included in the study. Arthrocentesis was performed as a minimally invasive therapeutic intervention in patients who had not responded adequately to at least six months of conservative management (medication, occlusal splint therapy, physiotherapy).

### Inclusion and exclusion criteria

Inclusion criteria were systemically healthy adults who provided informed consent and had intra-articular TMD persisting for ≥ 6 months despite conservative management (e.g., medication, splint therapy, and physiotherapy). Exclusion criteria were systemic rheumatologic diseases, bony or fibrotic ankylosis of the TMJ, previous open TMJ surgery, pregnancy or breastfeeding, and active infections.

### Clinical outcome assessments

All participants presented with unilateral TMJ involvement, and arthrocentesis was performed only on the affected side. For comparison, ultrasonographic capsular width measurements were obtained bilaterally from the arthrocentesis-treated (affected) and untreated (non-affected) sides.

All patients underwent a standardized TMJ arthrocentesis procedure. Ultrasonographic and clinical measurements were performed before and after treatment to evaluate changes in joint structure and mandibular function over time.

Capsular width was assessed using ultrasonography at three predefined time points: baseline (T0, pre-treatment), three months post-arthrocentesis (T1), and six months post-arthrocentesis (T2). These measurements were used to evaluate temporal changes in the articular capsule following the procedure.

Clinical assessments included MIO, pain intensity measured using the visual analog scale (VAS), lateral mandibular movements, and protrusive mandibular movement. MIO, lateral movements, and protrusion were measured in millimeters (mm) and recorded at T0, T1, and T2. Lateral mandibular movements were evaluated bilaterally on both the affected and non-affected sides, while protrusive movement was recorded to assess anterior mandibular translation.

Demographic characteristics (age and sex) and the side of arthrocentesis (only the symptomatic joint was treated; the non-affected side was considered as an untreated reference, allowing a within-subject comparison between the treated and untreated joints) were recorded to support subgroup and comparative analyses and to provide contextual interpretation of the structural and functional findings.

### Assessment and blinding

Outcome assessment was structured to minimize measurement bias. Pain intensity was self-reported by the patients using a standardized VAS, and the radiologist’s role in this process was limited to recording the reported scores.

All ultrasonographic evaluations and clinical outcome measurements, including MIO and mandibular movement assessments, were performed by a single radiologist who was independent of the surgical procedure, treatment planning, and postoperative patient management. The assessor also had no access to intraoperative findings at the time of measurement.

T0 ultrasonographic assessments were obtained prior to arthrocentesis, before any needle insertion or procedure-related tissue alteration, thereby providing an unmodified reference state. Follow-up ultrasonographic and clinical evaluations were conducted at T1 and T2 postoperatively using the same standardized measurement protocol.

All outcome measurements were obtained by an independent assessor using predefined criteria, which may have helped reduce the potential for measurement bias. However, given the longitudinal design of the study and the potential for observable changes in ultrasonographic and clinical parameters over time, complete blinding to the time points may not have been feasible, which should be considered when interpreting the findings.

### Surgical procedure

All arthrocentesis procedures were performed under local anesthesia in a standard clinical setting by a single experienced oral and maxillofacial surgeon. Prior to the intervention, each patient underwent a comprehensive clinical examination and ultrasonographic assessment to confirm suitability. After aseptic preparation of the preauricular region, local anesthesia was administered using 2 mL of articaine hydrochloride with epinephrine (Ultracain^®^ D-S forte). A conventional two-needle technique was employed to access the upper joint compartment. Two 18-gauge needles were inserted under anatomical guidance and joint flow verification. Once access was confirmed, the joint was irrigated with 150–200 mL of sterile Ringer’s lactate solution. The purpose of this lavage was to remove inflammatory mediators and intra-articular debris, thereby improving joint mobility and alleviating symptoms. During irrigation, gentle mandibular movements were performed to enhance joint decompression and disc alignment. No pharmacological agents were injected after the lavage. Post-procedure, all patients were instructed to follow a soft diet for one week and were prescribed non-steroidal anti-inflammatory drugs (NSAIDs) for pain management. Postoperative care also included avoiding excessive mandibular movements and adhering to the soft diet.

In patients with unilateral TMD, arthrocentesis was performed only on the symptomatic joint. Clinical parameters were assessed by categorizing each TMJ as affected or non-affected. This side-specific approach allowed for a clearer evaluation of treatment efficacy on the symptomatic joint.

### Ultrasonographic assessment

Evaluations of the articular capsule were performed using a high-resolution ultrasound device (Mindray DC-60, Nanshan, Shenzhen, China) equipped with a 14–16 MHz linear transducer (L46NE, 6 cm depth). The skin surface was cleansed with alcohol, and an adequate amount of ultrasound gel (Naturel Ultrason Gel, Turkey) was applied to ensure optimal acoustic transmission.

**Fig. 1:**
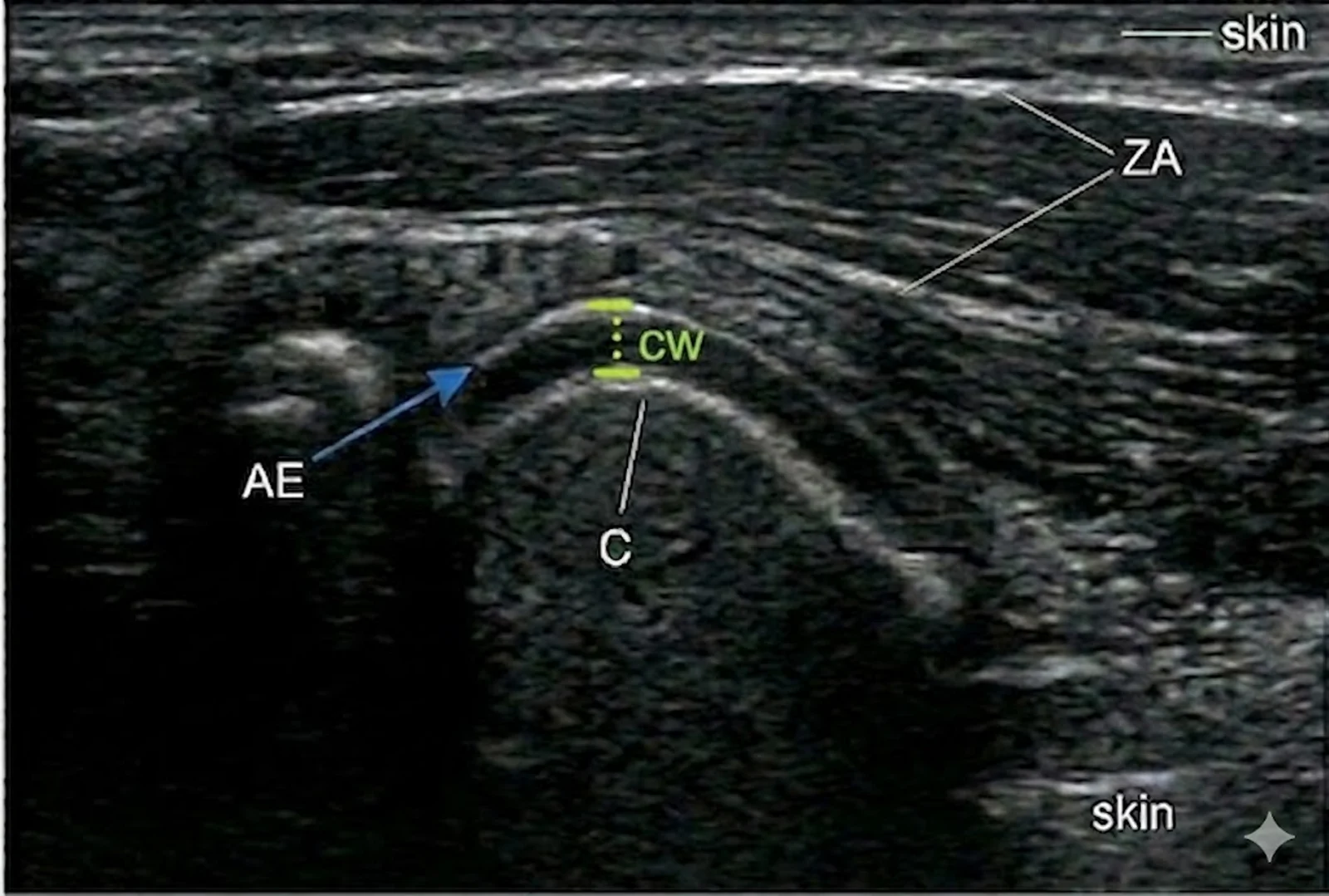
Ultrasonographic evaluation of the temporomandibular joint in the closed-mouth position. The ultrasound image displays the anatomical landmarks and the measurement line: skin surface, ZA (zygomatic arch), AE (articular eminence), and C (hyperechoic cortical outline of the laterosuperior surface of the condyle). The capsular width (CW, indicated by the green dotted line) represents the linear distance measured between the most superior points of the condylar outline and the hyperechoic capsular line

All examinations were performed with participants sitting upright and keeping their heads in a neutral position. First, the TMJ was localized by palpation during mouth opening and closing movements. After localization, the transducer was placed longitudinally along the zygomatic arch, and the mandibular condyle was identified in the closed-mouth position. The articular capsule was visualized as a well-defined hyperechoic band running parallel to the surface of the mandibular condyle. Capsular width was measured as the linear distance between this hyperechoic capsular line and the hyperechoic cortical outline of the laterosuperior surface of the condyle. Measurements were taken at the condylar level, connecting the most superior points of the two echogenic lines (Fig. 1). The distance between these boundaries corresponds to an indirect ultrasonographic indicator of intra-articular effusion and capsular width. To ensure consistency, each measurement was repeated twice, and the average value was recorded. Additionally, to assess intra-observer repeatability and control for individual measurement error, the same measurement set was repeated at 5-minute intervals during each session. All evaluations were performed by a single oral and maxillofacial radiologist with 12 years of clinical experience, using the same protocol and equipment at all time points (T0, T1, and T2).

### Statistical analyses

All statistical analyses were performed using SPSS software (version 21, IBM Corp., Armonk, NY, USA). Descriptive statistics were expressed as mean ± standard deviation (SD). Data normality was evaluated using the Shapiro–Wilk test. The Shapiro–Wilk test indicated that some variables (particularly change scores and follow-up VAS measurements) deviated significantly from normality (*p* < 0.05). Therefore, non-parametric tests were used for all analyses.

Between-group comparisons at T0 were performed using the Mann–Whitney U test. Changes in repeated measurements across three time points (T0, T1, and T2) were analyzed using the Friedman test. When significant differences were found, post-hoc pairwise comparisons were conducted using the Wilcoxon signed-rank test with Bonferroni correction. Intergroup comparisons between the affected and non-affected sides at each time point were performed using the Wilcoxon signed-rank test.

To evaluate associations between changes in capsular width and changes in clinical parameters (VAS, MIO, lateral and protrusive movements) from T0 to T2, Spearman’s rank correlation coefficients were calculated. A p-value < 0.05 was considered statistically significant.

The sample size was determined a priori using G*Power software (Version 3.1.9.7; University of Düsseldorf, Germany). The calculation was based on the morphometric parameters reported by Torres-Gaya et al. (2021), who evaluated spatial changes in capsular width during ultrasound-guided TMJ arthrocentesis in an anatomical study. They reported an approximately 3.0 mm difference in capsular width, with standard deviations of 2.0 mm for the operated side and 1.8 mm for the non-operated side. Using a two-tailed paired t-test with an alpha level of 0.05 and 80% statistical power (1–β = 0.80), the required minimum sample size was calculated to be 26 participants [15]. To account for potential dropouts and to increase reliability, 27 participants were included in the present study.

Due to the lack of clinically comparable prospective ultrasonographic studies evaluating longitudinal changes in capsular width after TMJ arthrocentesis, the reference study used for sample size estimation was ex vivo. To address this limitation, a post-hoc power analysis based on the observed effect size for the primary outcome (Cohen’s d = 1.42) was performed, indicating a statistical power of > 99%. However, it should be noted that post-hoc power calculations are based on observed effects and should therefore be interpreted with caution.

This study was conducted and reported in accordance with the STROBE (Strengthening the Reporting of Observational Studies in Epidemiology) Statement.

## Results

The study sample consisted of 27 subjects (16 females, 59.3%) with a mean age of 41.6 ± 13.0 years. Unilateral arthrocentesis was performed in 27 patients (Table 1).

**Table 1:** Bivariate analyses of the covariates vs. capsular width

Covariates	Capsular width-A	Capsular width-NA	*p*-value
	(Mean ± SD)	(Mean ± SD)	
Age	41.6 ± 13.0	41.6 ± 13.0	0.7*
Sex			
Female (*n* = 16)	2.1 ± 0.3	2.8 ± 0.3	0.3**
Male (*n* = 11)	2.0 ± 0.3	2.8 ± 0.4	
Side of the Arthrocentesis	n(%)	n(%)	n(%)
Unilateral	27(100)	0(0)	27(100)
Bilateral	0 (0)	0 (0)	0 (0)

*A* Affected, *NA* non-affected, *n* number of participants, *SD* Standard Deviation

*p* < 0.05

*Wilcoxon signed-rank test, **Mann–Whitney U test

The intra-observer reliability for capsular width measurements was excellent, with an intraclass correlation coefficient (ICC) of 0.94 (95% CI: 0.90–0.97).

Ultrasonographic measurements demonstrated a significant reduction in capsular width on the affected side over time, whereas no significant changes were observed on the non-affected side. The mean capsular width on the affected side decreased from 2.73 ± 0.29 mm at T0 to 2.46 ± 0.30 mm at T1, and 2.29 ± 0.33 mm at T2, and this change was statistically significant (*p* < 0.001). In contrast, the non-affected side showed relatively stable values across all time points (2.64 ± 0.25 mm, 2.64 ± 0.24 mm, and 2.66 ± 0.22 mm at T0, T1, and T2, respectively; *p* = 0.4). Between-group comparisons revealed no significant differences between sides at T0 (*p* = 0.3) or at T1 (*p* = 0.1). However, at T2, the affected side demonstrated a significantly smaller capsular width than the non-affected side (*p* < 0.001) (Table 2).

**Table 2:** Changes in capsular width over time

Time	Affected side (mm) Mean ± SD	Non-affected side (mm) Mean ± SD	p* value
T0	2.73 ± 0.29ᴬ	2.64 ± 0.25ˣ	0.3
T1	2.46 ± 0.30ᴮ	2.64 ± 0.24ˣ	0.1
T2	2.29 ± 0.33^C^	2.66 ± 0.22ˣ	**< 0.001**
p** value	**< 0.001**	0.4	

Different superscript letters indicate statistically significant differences between time points

T0: at baseline, T1: month 3, T2: month 6, mm: millimeter, SD: Standard Deviation

*p* < 0.05

*Wilcoxon signed-rank test, **Friedman test

The evaluation of clinical parameters demonstrated significant improvements over time following arthrocentesis (*p* < 0.001 for all variables). The mean VAS scores decreased markedly from 7.41 ± 1.55 at T0 to 3.56 ± 2.01 at T1, and 1.78 ± 1.37 at T2, indicating a substantial reduction in pain intensity over the follow-up period. The mean MIO increased significantly from 39.56 ± 10.15 mm at T0 to 43.85 ± 10.06 mm at T1, and 44.41 ± 11.85 mm at T2. Similarly, both lateral mandibular movements and protrusive movement exhibited progressive improvement. On the affected side, the mean lateral movement increased from 8.67 ± 2.63 mm at T0 to 9.37 ± 2.37 mm at T1, and 9.63 ± 2.37 mm at T2 (*p* < 0.001). On the non-affected side, values increased from 7.07 ± 3.08 mm at T0 to 8.89 ± 2.44 mm at T1, and 9.33 ± 2.29 mm at T2. The protrusive movement also improved significantly from 6.40 ± 2.50 mm at T0 to 7.50 ± 2.70 mm at T1, and 8.00 ± 2.90 mm at T2 (Table 3).

**Table 3:** Changes in clinical parameters over time following TMJ arthrocentesis

Variable	T0 Mean ± SD	T1 Mean ± SD	T2 Mean ± SD	p* value
VAS	7.41 ± 1.55^a^	3.56 ± 2.01^b^	1.78 ± 1.37^c^	**< 0.001**
MIO (mm)	39.56 ± 10.15^a^	43.85 ± 10.06^b^	44.41 ± 11.85^c^	**< 0.001**
LM - A (mm)	8.67 ± 2.63^a^	9.37 ± 2.37^b^	9.63 ± 2.37^c^	**< 0.001**
LM - NA (mm)	7.07 ± 3.08^a^	8.89 ± 2.44^b^	9.33 ± 2.29^c^	**< 0.001**
PM (mm)	6.40 ± 2.50^a^	7.50 ± 2.70^b^	8.00 ± 2.90^c^	**< 0.001**

Different superscript letters indicate statistically significant differences between time points

T0: at baseline, T1: month 3, T2: month 6, *TMJ* Temporomandibular joint, *VAS* Visual Analog Scale, *MIO* Maximum incisal opening, *LM* Lateral movement, *PM* Protrusive movement, *A* affected, *NA* Non-affected, *n* number of groups, *SD* Standard Deviation, *mm* millimeter

*p* < 0.05

*Friedman test

Spearman’s rank correlation analysis was performed to evaluate the relationship between the reduction in capsular width and improvements in clinical parameters from T0 to T2. A strong positive correlation was found between the change in capsular width (ΔCW) and the change in VAS scores (ΔVAS) on the affected side (*r* = 0.61, *p* < 0.001), indicating that greater reduction in capsular width was associated with greater pain relief. No significant correlations were observed between ΔCW and changes in MIO (*r* = -0.09, *p* = 0.65), lateral movement (*r* = -0.04, *p* = 0.84), or protrusive movement (*r* = -0.21, *p* = 0.29) (Table 4).

**Table 4:** Spearman correlation coefficients between changes in capsular width and changes in clinical parameters (T0 to T2)

Variable pair	*r* (Spearman)	*p* value
Δ Capsular width vs. Δ VAS	0.61	**< 0.001**
Δ Capsular width vs. Δ MIO	-0.09	0.65
Δ Capsular width vs. Δ LM	-0.04	0.84
Δ Capsular width vs. Δ PM	-0.21	0.29

Δ = Change from; T0: at baseline, T2: month 6; *VAS *Visual Analog Scale, *MIO *Maximum incisal opening, *LM* Lateral movement, *PM* Protrusive movement

## Discussion

This study aimed to evaluate the effects of TMJ arthrocentesis on capsular width, together with clinical parameters including pain intensity, MIO, and mandibular movements. The authors hypothesized that arthrocentesis would lead to symptomatic improvement and reduction in capsular width in patients with TMD. The findings demonstrated a significant decrease in capsular width on the affected side, along with reductions in VAS pain scores and increases in MIO as well as lateral and protrusive movements. These findings suggest that changes in capsular width may reflect alterations in intra-articular conditions, including pressure dynamics and possibly inflammatory status.

Arthrocentesis has been shown to reduce inflammation by removing intra-articular inflammatory mediators (e.g., IL-1β, IL-6, and TNF-α), thereby decreasing joint pressure and capsular width [16, 17]. More recent evidence also supports this mechanism and shows that arthrocentesis accelerates structural normalization by reducing joint inflammation [18]. Systematic reviews have emphasized its success in alleviating pain, restoring function, and decreasing intra-articular effusion [4, 19]. Collectively, the observed postoperative decrease in capsular width may be associated with a reduction in intra-articular inflammation and capsular distension, paralleling the observed symptomatic improvement. Consistent with previous reports, the current results may suggest that ultrasonographic measurement of capsular width is a potentially useful indicator for evaluating the therapeutic efficacy of arthrocentesis. Although a definitive threshold for clinically meaningful change in capsular width has not been established, the observed reduction was accompanied by significant improvements in pain and mandibular function. Taken together, these findings suggest that the magnitude of change may be considered clinically relevant, particularly when interpreted in conjunction with functional outcomes. This interpretation is further supported by the significant correlation observed between the reduction in capsular width and the decrease in VAS scores. However, it should be emphasized that ultrasonographic capsular width is an indirect parameter and cannot be considered a definitive measure of intra-articular inflammation.

A previous study reported that the capsular width was significantly greater in painful TMJs (2.04 ± 0.49 mm) than in pain-free joints (1.37 ± 0.41 mm) and that there was a positive correlation between the capsular width and pain intensity [20]. Similarly, another study demonstrated a significant decrease in capsular width and VAS scores during the 3-month follow-up after arthrocentesis [21]. In addition, another study reported that modified arthrocentesis techniques led to normalization of joint morphology by reducing intra-articular inflammation and capsular tension [18]. Furthermore, multiple clinical trials have confirmed that arthrocentesis reduces inflammation and significantly improves VAS, MIO, and lateral mandibular movement parameters [22–25]. Collectively, these findings support the notion that arthrocentesis promotes clinical improvement through both the biochemical removal of inflammatory mediators and mechanical enhancement of joint mobility. The present study’s observation of capsular width reduction on the affected side is consistent with previous evidence suggesting that capsular width may serve as an indirect indicator of intra-articular effusion and may be associated with inflammatory changes.

The strengths of this study include its prospective design, simultaneous evaluation of clinical and ultrasonographic data, and the use of objective, quantitative measurements. Furthermore, the use of standardized DC/TMD ensured a homogeneous patient population with confirmed intra-articular pathology, thereby reducing the risk of selection bias.

However, several limitations should be considered. The relatively small sample size and short follow-up period may limit the generalizability of the findings. In addition, ultrasonographic measurements may be subject to inter-observer variability, given the operator-dependent nature of the technique.

Another important limitation is that the non-affected side was used as an untreated reference for comparison. Although all participants presented with unilateral clinical symptoms, subclinical or bilateral involvement of TMD cannot be completely excluded. Therefore, the non-affected side may not represent a truly healthy control, and the findings should be interpreted as a within-subject comparison rather than a case–control design.

Moreover, although the arthrocentesis procedure was performed using a standardized protocol, minor variations in technique—such as the extent of mandibular manipulation during lavage—may have occurred. These subtle differences could have influenced the outcomes to some extent and should be taken into account when interpreting the results.

Finally, despite its advantages, ultrasonography has inherent limitations. It is an operator-dependent imaging modality, and its diagnostic accuracy may vary depending on the examiner’s experience. In addition, it has limited capability in visualizing deeper intra-articular structures compared to magnetic resonance imaging, which is considered the gold standard for soft tissue evaluation of the temporomandibular joint. As no direct validation with MRI was performed in this study, the ultrasonographic findings should be interpreted with caution.

## Conclusion

This study shows that TMJ arthrocentesis is associated with improvements in pain and mandibular function, along with a significant reduction in articular capsular width. The reduction in capsular width may reflect changes in intra-articular conditions, including a possible reduction in inflammation, and may represent a potential structural marker of joint recovery. Notably, the observed reduction in capsular width was significantly correlated with the decrease in pain intensity, supporting its potential clinical relevance. Based on these findings, ultrasonographic assessment of capsular width could serve as a non-invasive and objective tool for monitoring the therapeutic efficacy of arthrocentesis. Future long-term, controlled studies with larger sample sizes are warranted to further clarify the relationship between changes in capsular width and clinical outcomes.

## Data Availability

The datasets used and/or analyzed during the current study are available from the corresponding author on reasonable request.
